# Photoreceptor spectral tuning by colorful, multilayered facet lenses in long-legged fly eyes (Dolichopodidae)

**DOI:** 10.1007/s00359-016-1131-y

**Published:** 2016-11-21

**Authors:** D. G. Stavenga, A. Meglič, P. Pirih, H. Koshitaka, K. Arikawa, M. F. Wehling, G. Belušič

**Affiliations:** 1 Computational Physics, Zernike Institute for Advanced Materials, University of Groningen, NL9747AG Groningen, The Netherlands; 2 Biotechnical Faculty, University of Ljubljana, Večna pot 111, 1000 Ljubljana, Slovenia; 3 Laboratory of Neuroethology, Sokendai-Hayama, The Graduate University for Advanced Studies, Hayama, 240-0193 Japan; 4 Air Force Research Laboratory, Eglin Air Force Base, FL 32542-6810 USA

**Keywords:** Iridescence, Visual pigments, Spectral filters, Spectral sensitivity, Photoreceptors

## Abstract

The facet lenses of the compound eyes of long-legged flies (Dolichopodidae) feature a striking, interlaced coloration pattern, existing of alternating rows of green-yellow and orange-red reflecting facets, due to dielectric multilayers located distally in the facet lenses (Bernard and Miller. Invest Ophthalmol 7:416–434 (1968). We investigated this phenomenon in the dolichopodid *Dolichopus nitidus* by applying microspectrophotometry, electron microscopy and optical modeling. The measured narrow-band reflectance spectra, peaking at ~540 and ~590 nm with bandwidth ~105 nm, are well explained by a refractive index oscillating sinusoidally in six periods around a mean value of about 1.44 with amplitude 0.6. The facet lens reflectance spectra are associated with a spectrally restricted, reduced transmittance, which causes modified spectral sensitivities of the underlying photoreceptors. Based on the modeling and electroretinography of the dolichopodid *Condylostylus japonicus* we conjecture that the green and orange facets narrow the spectral bandwidths of blue and green central photoreceptors, respectively, thus possibly improving color and/or polarization vision.

**Electronic supplementary material:**

The online version of this article (doi:10.1007/s00359-016-1131-y) contains supplementary material, which is available to authorized users.

## Introduction

When observing insect compound eyes with a microscope, the most prominent property is the lattice of facet lenses, which mark the ommatidia, the eyes’ visual units. The facet lenses focus incident light from the environment on the photoreceptor cells, which transduce the absorbed light into an electrical signal, thus starting the visual process. To warrant a limited directional sensitivity of the photoreceptor cells, they are commonly surrounded by pigment cells (Land and Nilsson 2002).

Sensibly, the facet lenses are virtually always fully transparent in the visual wavelength range, which extends well into the ultraviolet (UV), but the facet lens transmittance starts to drop at very short wavelengths, below 350 nm (Miller 1979; Knüttel and Lunau 1997; Ilić et al. 2016). Interestingly, in some insect species, specifically in various dipteran families, the facet lenses have also a reduced transmittance in the visible wavelength range, which can be recognized in the intact eye from a distinctly colored cornea. Specifically, many tabanid and dolichopodid fly species have beautifully colored facet lenses, with narrow-band reflectance spectra (Bernard and Miller 1968, 1970; Bernard 1971; Miller 1979). The distinct reflectance is accompanied by a reduced transmittance (Friza 1929; Lunau and Knüttel 1995; Knüttel and Lunau 1997; Leertouwer and Stavenga 2000; Stavenga 2002). A question that immediately arises is how this might function to improve vision.

Half a century ago, Bernard and Miller performed transmission electron microscopy on horsefly eyes, and thus demonstrated that the outermost part of the facet lenses contains a stack of ~100 nm thick layers, with alternatingly high and low electron density (Bernard and Miller 1968; Miller 1979). To explain the observed reflection phenomena, they performed optical modeling, applying dielectric multilayer theory. Assuming refractive index values 1.7 and 1.45 for the alternating layers they concluded that the multilayered facet lenses thus will act as effective spectral filters for the ommatidia’s photoreceptor set. We have to note, however, that the refractive index of insect chitinous cuticle is distinctly lower than 1.7 (Leertouwer et al. 2011), hence necessitating a closer look at the modeling results.

In a remarkable follow-up study, Bernard (1971) performed extensive microspectrophotometry on dipteran flies with shiny facets, including horseflies, deerflies, a soldierfly and fruitfly, as well as many long-legged flies (Dolichopodidae). In the tabanids, notably the females have often colorful, wavy bands of facets or dotted patterns, characteristic for the species, but the dolichopodids with their intertwined, alternating colored rows of facets have even more stunning colored facet patterns (Bernard and Miller 1968; Trujillo-Cenóz and Bernard 1972).

The extreme ordering of the colored facets of dolichopodids is probably related to the organization of their visual system (Trujillo-Cenóz and Bernard 1972). Generally, dipteran flies have two main classes of photoreceptors, the peripheral or outer photoreceptors R1–6 and the central or inner photoreceptors R7 and R8 (Hardie 1985; Behnia and Desplan 2015). The R1–6 serve motion vision, while the R7 and R8 participate particularly in color vision, although R1–6 may contribute (Fukushi 1989; Troje 1993; Morante and Desplan 2008; Yamaguchi et al. 2010; Wardill et al. 2012; Schnaitmann et al. 2013; Garbers and Wachtler 2016). The R7 and R8 photoreceptors thus are the prominent candidates for improved spectral discrimination by selective spectral filtering.

Extensive research on the anatomy, visual pigments and spectral sensitivity of the central photoreceptors in higher brachyceran dipterans, specifically the housefly *Musca domestica*, the fruitfly *Drosophila melanogaster*, and the blowfly *Calliphora vicina*, has revealed that there are two main classes of ommatidia with different sets of R7 and R8 receptors. The two ommatidial classes are randomly distributed in the main part of the eye and distinguished as p (pale) and y (yellow), because of the discovery that the R7 rhabdomeres of the y-class of *Musca* contain strongly blue-absorbing carotenoid pigment, causing the yellow color of the central rhabdomeres when observing retinal sections in transmission light microscopy; the central rhabdomeres of the p-class ommatidia are pale (Kirschfeld and Franceschini 1977). Spectral sensitivity measurements revealed that all R7 receptors of *Musca* are mainly sensitive in the UV, while the R8 in p-class ommatidia are blue receptors and the R8 in y-ommatidia are green receptors (Hardie 1985). Detailed anatomy of the R7 and R8 rhabdomeres in *Calliphora* blowflies revealed a distinct difference in orientation of the microvilli, depending on the ommatidial class (Wunderer and Smola 1982).

Interestingly, the different rows of colored facets of the dolichopodids, which belong to the lower brachycerans (Marshall 2006), mark ommatidia with differently organized central rhabdomeres. Trujillo-Cenóz and Bernard (1972) found that the dolichopodid *Sympycnus lineatus* has alternating rows of yellow and red facets corresponding to the different microvillar organization of especially the central photoreceptors. In all ommatidia, the microvilli of R8 were oriented dorsoventrally, i.e., parallel to the eye’s vertical axis. In yellow-facetted ommatidia, the microvilli of R7 were oriented perpendicular to those of R8, while in the ommatidia with red facets the microvilli of R7 were parallel to those of R8. Trujillo-Cenóz and Bernard (1972) noted that the central photoreceptors with vertical microvilli will be partially blind to horizontally polarized surface glare and they, therefore, speculated that these cells might have a specific function in detecting prey against reflecting surfaces.

Here we investigated the eyes of the long-legged fly *Dolichopus nitidus*, which has eyes with alternating rows of green and orange facets, by combined microspectrophotometry, anatomy, and optical modeling. We show that the two facet types consist of very similar multilayers, only slightly differing in layer thicknesses. The possible filter functions of the facet multilayers are discussed.

## Materials and methods

### Animals

Long-legged flies, *Dolichopus nitidus* and *Condylostylus japonicus*, were captured on plant leaves near the Sokendai campus in Hayama, Japan.

### Spectrophotometry

The reflectance spectra of individual facets of the eyes of *Dolichopus nitidus* were measured with a microspectrophotometer (MSP), being a Leitz Ortholux microscope (Leitz, Wetzlar, Germany) connected to an AvaSpec 2048-2 CCD detector array spectrometer (Avantes, Apeldoorn, Netherlands), with light supplied by a xenon arc light source. The microscope objective was an Olympus LUCPlanFL N 20×/0.45. As a reference, a white diffuse reflectance tile (Avantes WS-2) was used, which, however, causes overestimated reflectance spectra, because the multilayers in the facet lenses reflect directionally, while the reference tile reflects diffusely. We, therefore, divided the measured spectra by a factor 3, estimated as the approximate factor of overestimation. The size of the measured area was 10 × 10 µm^2^.

### Anatomy

The fine-structure of the facet lenses was studied with transmission electron microscopy. Eyes were first separated from the body and subsequently treated with standard procedures (fixated for 3 h in 4% paraformaldehyde and 3.5% glutaraldehyde, dehydrated in ethanol series, incubated 90 min in 0.1 M OsO_4_ in 0.1 M Na-cacodylate, pH 7.4, and embedded in Spurr resin). Ultrathin sections were made with a diamond knife and observed with a Philips CM100 electron microscope.

### Optical modeling

We used the transmission electron micrographs for modeling the reflectance spectra of the cuticle and facet lenses with a transfer matrix formalism for dielectric multilayers (Stavenga 2014). We first selected in the micrographs 5 lanes (width 0.5 µm), and then determined in 10 nm thick cross-sections the average optical density. As the material of cuticle and facet lenses is most likely a mixture of chitin and water (Miller 1979), the refractive index values of cuticle and facet lenses are hence restricted to lie between the refractive indices of pure chitin and pure water (note: chitin indicates here fully dry cuticle). The wavelength dependence of the refractive index of chitin is well approximated by the Cauchy formula *n*_c_ = *A*_c_ + *B*_c_λ^−2^, with *A*_c_ = 1.517 and *B*_c_ = 8800 nm^−2^ (Leertouwer et al. 2011); the refractive index of water is well described by *n*_w_ = *A*_w_ + *B*_w_λ^−2^, with *A*_w_ = 1.325 and *B*_w_ = 3093 nm^−2^ (see the tabled data of Daimon and Masumura 2007, or those in http://www.philiplaven.com/p20.html). We determined the maximum and minimum values of the density profiles and assumed that the refractive index value there was that of pure chitin or water, respectively. Assuming that the refractive index is proportional to the density, we then converted the intermediate density values into refractive index values by interpolation. With the obtained refractive index profiles, we finally calculated the reflectance spectrum of each lane.

The assumption that the maximal and minimal density values of the multilayers in the micrographs are due to pure chitin and water is most probably too extreme, and, therefore, we investigated the consequences of more moderate refractive index contrasts by considering various multilayers sandwiched in between air, refractive index *n*_a_ = 1, and a medium consisting of equal parts of chitin and water, i.e., with refractive index *n*_*f*_ = (*n*_c_ + *n*_w_)/2. The refractive index of the multilayers was taken to vary sinusoidally in the distal part of the lens as given by $n(z) = n_{f} + \Delta n\sin (2\pi z/d),$ with amplitude $\Delta n = f(n_{c} - n_{w} )/2$, where *z* is the depth coordinate, *d* the period length, and *f* the modulation parameter. We chose *d* = 200 nm and 6 values for *f*: 0.0, 0.2, 0.4–1.0; if *f* = 1.0, the maximum and minimum refractive index values, *n*_c_ and *n*_w_, are reached at *d*/4 and 3*d*/4. We explored the effect of a variable layer number by calculating the reflectance spectra for multilayered structures with 4, 6 and 8 periods of the sinusoidally oscillating refractive index.

### Electroretinography

The spectral sensitivity of dorsal and ventral areas of the eyes of *Condylostylus japonicus*, both female and male, was measured by recording electroretinograms, using glass pipettes filled with tap water as recording electrodes. Light stimuli were provided by a 500 W xenon lamp via a series of narrow-band interference filters ranging from 300 to 740 nm (for details, see Wakakuwa et al. 2014).

## Results

### The dolichopodid eye’s interlaced reflection pattern

**Fig. 1:**
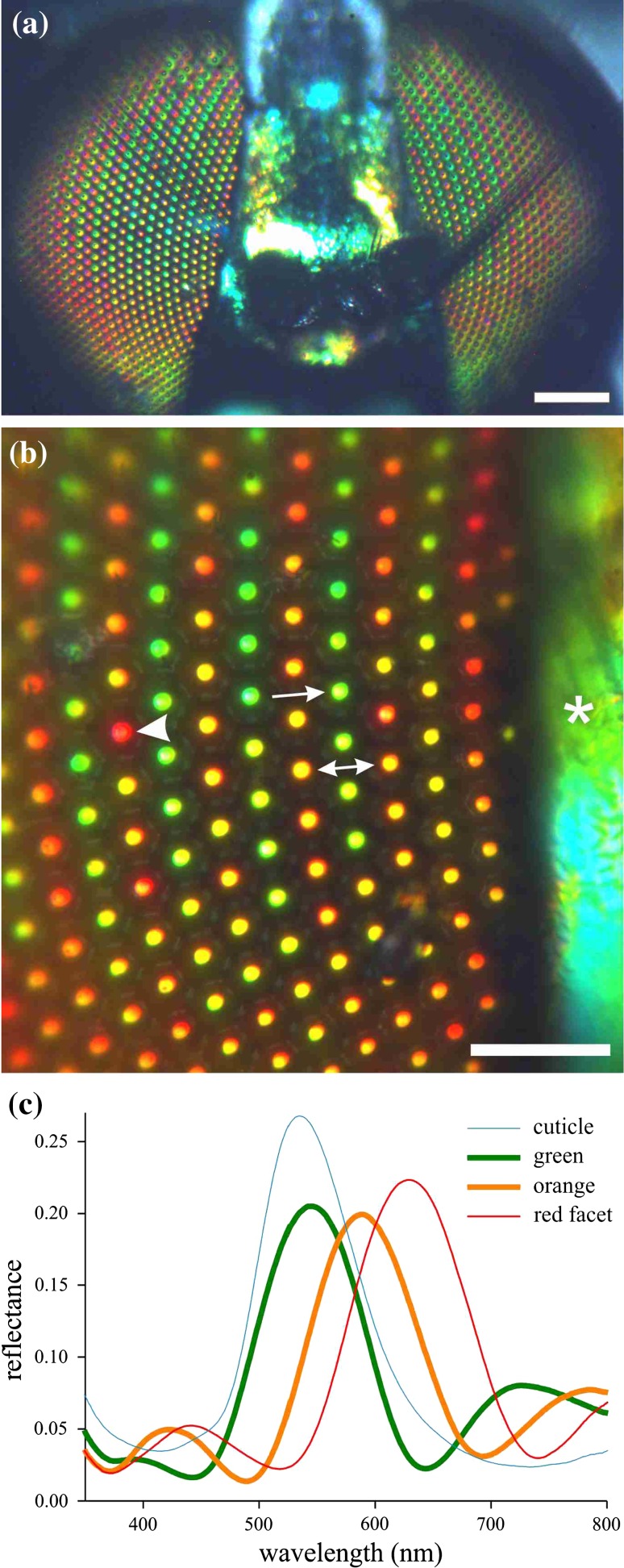
Shiny facets and cuticle of the dolichopodid *Dolichopus nitidus* and associated reflectance spectra. **a** The eyes are about symmetrical concerning the arrangement of* colored* facets. **b** Close-up view showing interlaced rows with* green* (*arrow*) and* orange* (*double-headed arrow*) facets, with also an occasional* red* facet (*arrowhead*). The cuticle (*asterisk*) reflection is* green*. **c** Reflectance spectra of a* green* (*arrow*, **b**),* orange* (*double arrow*) and* red* (*arrowhead*) facet and cuticle (*asterisk*) measured by MSP. *Scale bars* **a** 200 µm, **b** 50 µm

The eyes of *Dolichopus nitidus* observed with epi-illumination show a regular pattern with rows of alternating green and orange reflecting facets. The pattern of both eyes is virtually symmetrical (Fig. 1a). Quite noticeably, the facets in a row have not exactly the same color, and for instance in rows of orange facets occasional red colored facets occur (Fig. 1b). Reflectance spectra measured from the individual facets with a microspectrophotometer have distinct bands with peak wavelengths at ~540 and ~590 nm and a very similar bandwidth of ~105 nm (Fig. 1c). In fact, the spectra also have similarly oscillating side bands and appear as being simply shifted along the wavelength scale, clearly indicating a similar optical basis (see also Supplementary Fig. S1).

The dolichopodid’s body cuticle exhibits a clear metallic reflection (Fig. 1a, b). For instance, the head cuticle in between the eyes is greenish. Its reflectance spectrum resembles that of the facet lenses but is slightly broader and lacks the side bands (Fig. 1c).

### Anatomy of cuticle and facet lenses

**Fig. 2:**
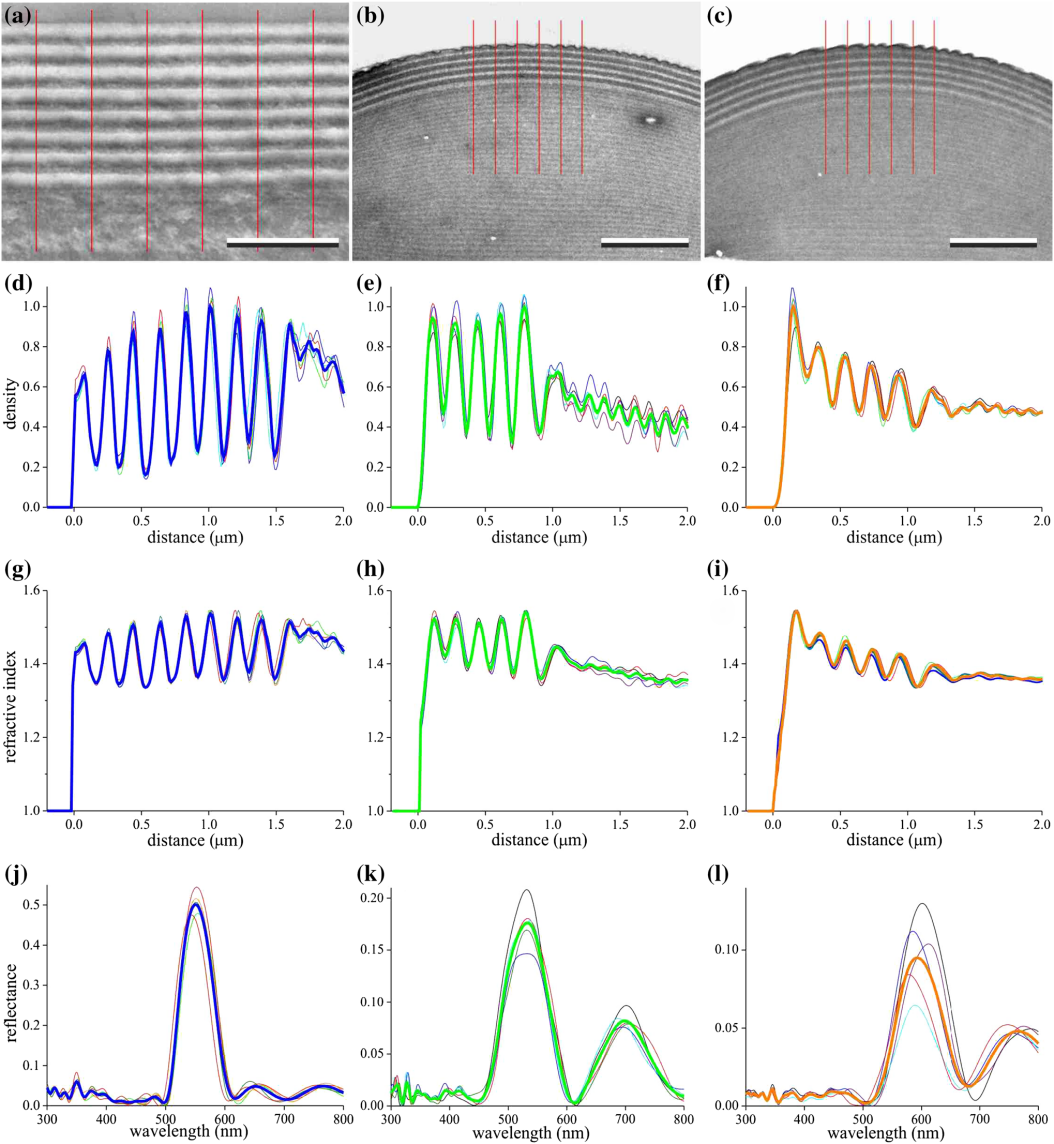
Transmission electron microscopy and modeled reflectance spectra of head cuticle and facet lenses. **a**–**c** Local sections of the head cuticle (**a**) and the two types of facet lenses (**b**, *green*; **c**, *orange*). *The red lines mark lanes* with 0.5 µm width;* bars* **a** 1 μm, **b** and **c** 2 μm. **d**–**f** Density profile of the part of the 5 lanes of (**a**–**c**) normalized to the maximum of the mean profile (*bold curves*). **g**–**i** Refractive indices (at 550 nm) derived from the density profiles of (**d**–**f**) by assuming that the refractive index is proportional to the density and is restricted to the range between the refractive indices of chitin and water. **j**–**l** Reflectance spectra for normal illumination calculated with the refractive index profiles of (**g**–**i**); *bold curves* averaged spectra

To understand the reflectance spectra quantitatively, we performed transmission electron microscopy (Fig. 2a–c). Sections perpendicular to the cuticle and facet lens surfaces demonstrated distal multilayered structures. The period length of the density fluctuations in the cuticle was ~190 nm (Fig. 2a). We encountered generally two types of facets with length of the layer period ~180 nm (green facets, Fig. 2b) and ~200 nm (orange facets, Fig. 2c); Supplementary Figure S2 presents a micrograph with the transition area of the two facet types.

We determined the mean electron density of five adjacent lanes, each 0.5 µm wide, of the micrographs (Fig. 2a–c) in 10 nm thick slices, and subsequently divided the density functions by the maximum of the average density function (Fig. 2d–f). The density profile of the cuticle had ~8–9 peaks and valleys (Fig. 2d), but for the facet lenses there were ~5–6 peaks and valleys (Fig. 2e, f). The facet lenses showed at the outer surface minor nipples, the effect of which is a small reduction of the reflectance (Stavenga et al. 2006), but this can be fully neglected with respect to the major effect of the multilayers on the reflectance.

### Modeling the reflectance of the multilayers in the cuticle and facet lenses

The varying electron density of the transmission electron micrographs revealed a layered structure, presumably functioning as a dielectric multilayer. We converted the obtained density profiles (Fig. 2d–f) into refractive index profiles (Fig. 2g–i), and then calculated reflectance spectra with a matrix transfer formalism (see Materials and methods).

The refractive index profiles of the five lanes in the cuticle micrograph yielded almost identical reflectance spectra with a major reflectance band, peak wavelength 535 nm and halfwidth 60 nm; the measured spectra had a peak wavelength of ~550 nm and halfwidth of ~100 nm (Fig. 1c). This difference can be readily explained by considering that the width of the very thin TEM section of Fig. 2a is only a few μm, while the measured reflectance spectra are from an area with size 100 μm^2^. The multilayer thicknesses will not remain constant over lateral distances of several μm, which hence will result in a broadened reflectance band. However, this does not explain adequately that the calculated reflectance peak value of ~0.5 is much larger than the measured value ~0.25. A most likely reason for the difference is that the amplitude of the refractive index oscillations used in the calculations has been overestimated.

The density profiles of the facet lens micrographs differed somewhat (Fig. 2e, f), presumably due to the variability in staining and photographic processing. The converted refractive index profiles of the five lanes (Fig. 2h, i) yielded reflectance spectra with somewhat variable shape and amplitude (Fig. 2k, l).

**Fig. 3:**
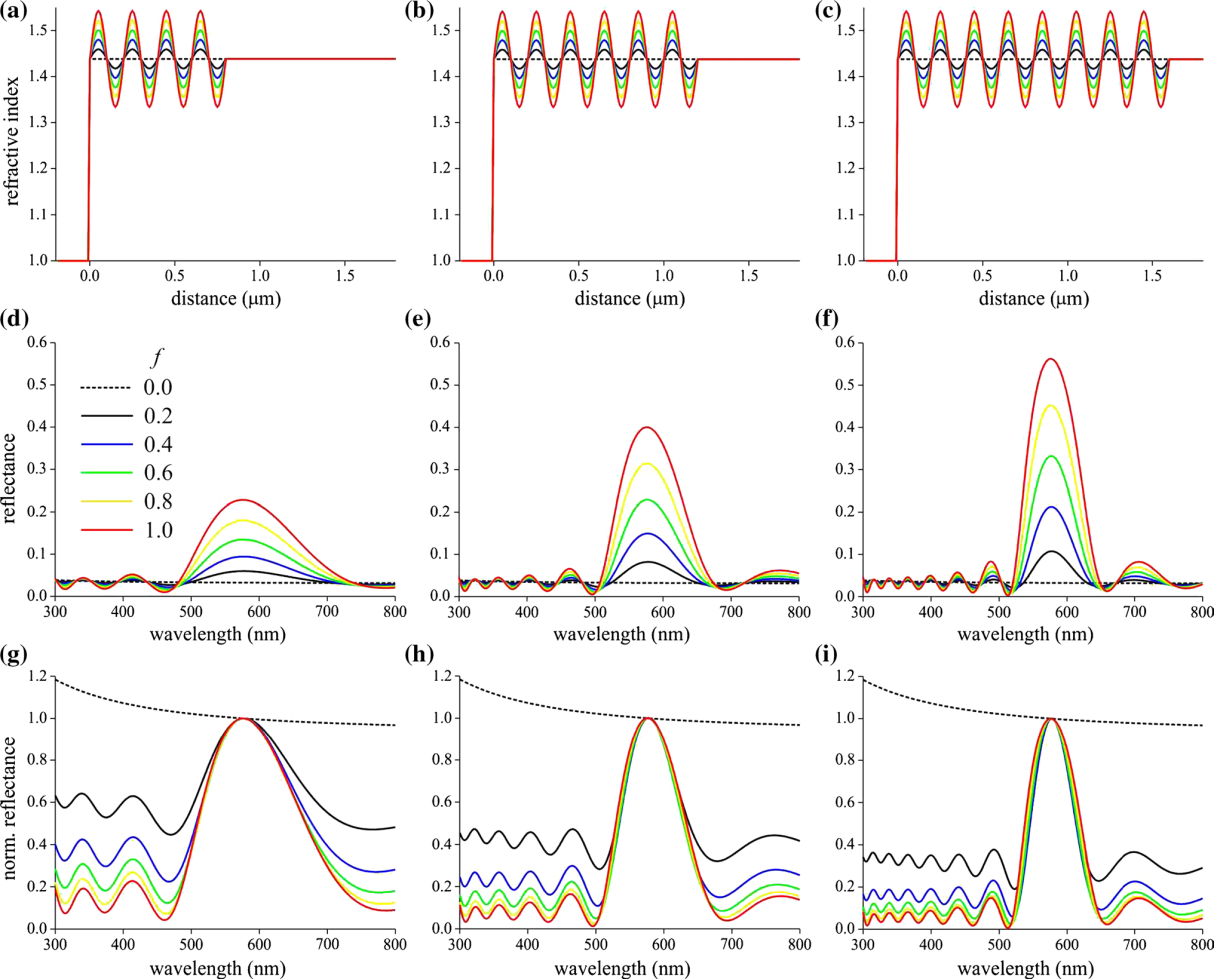
Modeling reflectance spectra of facets with a distal multilayer structure. **a**–**c** Refractive index profile (at 576 nm) of a slab with sinusoidally oscillating refractive index with period length 200 nm in between air and a medium with cuticle/water ratio 1 and modulation factor *f* = 0.0, 0.2, 0.4–1.0. **d**–**f** Corresponding reflectance spectra (normal illumination). **g**–**i** The spectra of (**d**–**f**) normalized at the peak wavelength, 576 nm. **a**, **d**, **g** slab with 4 periods; **b**, **e**, **h** 6 periods; **c**, **f**, **i** 8 periods. The *dotted lines* represent cases where the refractive index is not oscillating

Because of the uncertainties in the exact physical values of the multilayers, we investigated a model system consisting of a slab with sinusoidally oscillating refractive index in between air and a medium with chitin/water ratio 1 (see “Materials and methods”). We chose as the period length *d* = 200 nm, the number of periods 4, 6, and 8, and for the modulation factor of the oscillating refractive index *f* = 0.0, 0.2, 0.4, 0.6, 0.8, and 1.0 (Fig. 3a–c). The reflectance spectra calculated for the various cases have a number of prominent features (Fig. 3d–f). First, the reflectance peak value rapidly increases with increasing period number. Furthermore, the bandwith decreases, which is most clearly seen when normalizing the spectra to the reflectance value at the peak wavelength, 576 nm (Fig. 3g–i).

The case *f* = 0 represents a continuous medium with refractive index *n*_*f*_ = (*n*_c_ + *n*_w_)/2 facing air, where *n*_c_ and *n*_w_ are the refractive indices of chitin and water. Due to the dispersion of the refractive indices, the reflectance value (~0.03; Fig. 3d–f, dashed curves) is slightly wavelength dependent, recognizable from the normalized spectra (Fig. 3g–i, dashed curves). The reflectance spectra of the other cases, when *f* > 0, oscillate outside the main reflectance band around the value ~0.03.

The shape of the modeled reflectance spectra (Fig. 3g–i) closely resembles that of the measured spectra (Figs. 1c, S1). The measured facet lens spectra have peak values ~0.2. From the spectra of Fig. 3e, i.e. for a multilayer with six periods (as follows from the anatomy, Fig. 2b, c), we then derive that the modulation parameter of the varying refractive index of the facet lenses is *f* ≈ 0.6, or, that the refractive index oscillates between 1.38 and 1.50, with mean 1.44 (at 576 nm).

### Transmittance of the facet lenses and photoreceptor spectral sensitivity

**Fig. 4:**
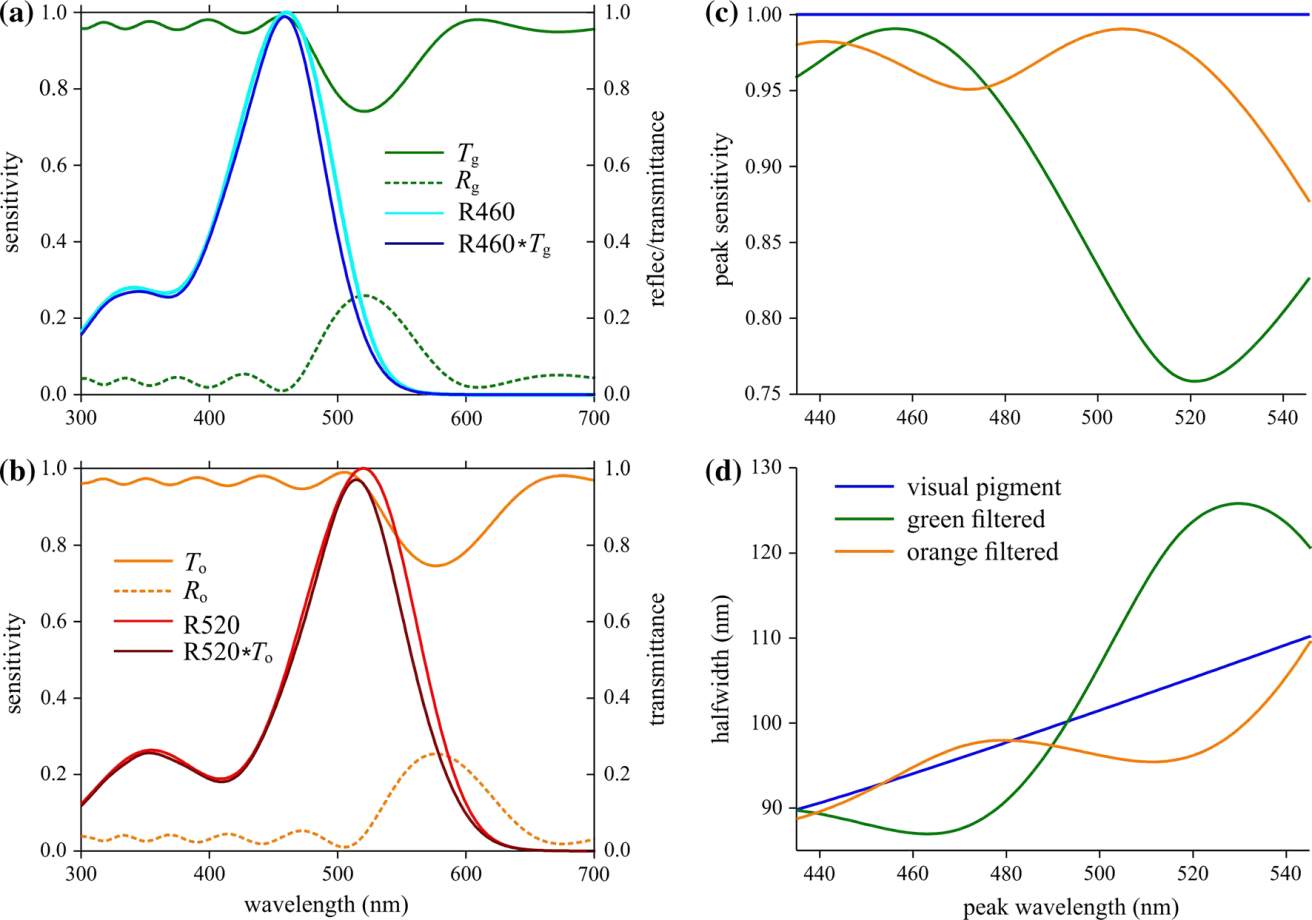
Modification of photoreceptor spectral sensitivities by the green and orange corneal filters having reflectances *R*_g_ and *R*_o_ and transmittances *T*_g_ and *T*_o_. **a** Rhodopsin R460 (peak wavelength 460 nm) filtered by the green reflector (*T*_g_) yields a slightly shifted sensitivity spectrum, but the orange reflector has negligible effect. **b** Rhodopsin R520 (peak wavelength 520 nm) filtered by the orange reflector (*T*_o_) yields a slightly shifted sensitivity spectrum. **c** Peak sensitivity of a receptor due the two spectral filters of (**a**) and (**b**). **d** Halfwidth of the sensitivity spectra of these receptors

The distinct reflectance of the facet lenses causes a reduced transmittance, and hence the facet lens will act as a spectral filter. We have studied the spectral filtering for facet lenses with model multilayers with six periods of oscillating refractive indices, as those in Fig. 3b. We assumed a modulation parameter *f* = 0.6 and considered two cases with period length 180 and 200 nm (to match the period lengths of layers in green and orange facets, respectively), yielding reflectance spectra with peak wavelengths 520 and 576 nm, indicated in Fig. 4a, b by *R*_g_ (green) and *R*_o_ (orange). As the absorption of chitin and water in the visible wavelength range is negligible, the facet lens transmittance spectra are immediately derived from the reflectance spectra by *T* = 1–*R*. We heuristically investigated the spectral effects of the transmittance spectra, *T*_g_ and *T*_o_ (Fig. 4a, b).

Figure 4a shows the effect of the green facet lens filter (*T*_g_) on a blue receptor with a spectral sensitivity equal to that of a rhodopsin peaking at 460 nm (R460). The filter reduces the peak sensitivity negligibly and slightly narrows the spectral bandwidth (Fig. 4a). The effect of the orange filter (*T*_o_) on a green receptor with spectral sensitivity equal to that of rhodopsin peaking at 520 nm (R520) is a minor reduction of the peak sensitivity and a noticeable narrowing of the spectral bandwidth (Fig. 4b). The effects of the two filter types on the peak sensitivity and bandwith of receptors with various rhodopsins are shown in Fig. 4c, d. Generally, when the rhodopsin peak wavelength is substantially separated from the filter peak wavelength, the peak sensitivity is hardly affected. The two filters noticeably narrow the spectral sensitivity bandwidth when the rhodopsin peak wavelength is ~470 and ~525 nm, respectively, i.e. when the peak wavelengths of the filter and the rhodopsin differ ~50 nm. The green filter has rather adverse effects on green rhodopsins, as it reduces the peak sensitivity and broadens the bandwidth (Fig. 4c, d).

Generally, modeling shows that spectrally shifted filters have most noticeable effects on rhodopsins with peak wavelengths ~50 nm shorter than the peak of the corneal filter, meaning that red facets with peak wavelengths well above 600 nm will not play a role as spectral filters, as fly visual pigments with peak wavelengths >550 nm are not known to exist.

## Discussion

### Color patterns in the eyes of dolichopodids

The eyes of *Dolichopus nitidus* are marked by rows of green and orange facets, with reflectance peak wavelengths around 540 and 590 nm, respectively. Facets with intermediate colors and thus intermediate peak wavelengths amply exist, and also occasional facets with much red-shifted spectra occur, but the spectral shape is always virtually identical (Figs. 1, S1). Spectra with similar shape, although narrower bandwith were reported by Bernard (1971) for dolichopodid flies of the genus *Condylostylus*. The reflectance spectra of the corneal facets, and hence the colors, can substantially differ, dependent on the dolichopodid species and sex (Bernard 1971). In some cases, the distinct interlaced pattern of alternating green-yellow and orange-red facet rows abruptly shifts, as shown in Supplementary Figure S3; see also https://www.nyu.edu/projects/desplan/Members/mike/mike.html.

### Refractive index of fly facet lenses

In the absence of a multilayer, the reflectance of a facet lens with refractive index *n*_*f*_ = (*n*_c_ + *n*_w_)/2 facing air is for normal illumination according to Fresnel’s equations [(*n*_*f*_ – *n*_*a*_)/*n*_*f*_ + *n*_*a*_)]^2^=3–4% (Fig. 3d–f; *f* = 0). Adding a distal multilayer can considerably augment the reflectance in a restricted wavelength range, depending on the optical pathlength of the multilayer’s period. Outside the main reflectance peak, the reflectance oscillates around the reflectance value of a facet without muiltilayers, i.e. when *f* = 0 (Fig. 3d–f).

We calculated reflectance spectra of the differently colored facet lenses using refractive index profiles calculated from the density of transmission electron micrographs. The refractive index profiles indicated that the refractive index decreases with the depth into the facet lenses, in line with an interference microscope study on slices of the facet lenses of the blowfly *Calliphora* showing that the refractive index decreases from 1.473 to 1.415, going from distally to proximally (Seitz 1968). A strongly graded refractive index was reported by Vogt (1974) for the facet lenses of the moth *Ephestia kühniella*, which reaches at the corneal surface a refractive index value 1.55, declining to 1.43 at the proximal basis. Other previous studies on the facet lenses of higher dipterans reported for the housefly *Musca* ~1.44 (McIntyre and Kirschfeld 1982) and the blowflies *Calliphora* and *Chrysomia* 1.43 and 1.40 (Stavenga et al. 1990). These values approximate the refractive index of a medium consisting of about equal amounts of chitin and water. We assumed this to be the case for the main dolichopodid facet lens medium in the modeling of Fig. 3. Bernard considered discrete multilayers, with refractive index values 1.7 and 1.45 for the alternating layers (Bernard and Miller 1968) and also 1.65 and 1.34, with 1.473 for the main lens medium (Fig. 23 in Miller 1979). The normalized reflectance spectra thus calculated well-fitted the normalized reflectance spectra measured in the long-legged fly species *Condylostylus*. This study suggests that the multilayers rather have an about sinusoidally varying refractive index with a moderate amplitude of 0.06, or, with a refractive index for the main lens medium ~1.44, the upper limit is ~1.50 and the lower bound ~1.38.

In the modeling we investigated the reflectance spectra of 4, 6 and 8 periods, with sinusoidally varying refractive index, because the micrographs showed ~8 (head cuticle) or 5–6 (facets) dense and dilute layers (Fig. 2); a similar number was found for the facet lenses of *Condylostylus* by Bernard (1971). The reflectance spectra calculated with a modulation factor *f* = 0.6 best approximated the measured spectra. The variation in peak wavelength of the reflectance spectra measured from the differently colored facets indicates that the multilayer period varies within one facet class, although by no more than ~20%.

### Reflectance and transmittance spectra of fly facet lenses

The absolute value of the facet lens reflectance spectra could not be accurately determined due to a few technical uncertainties. For instance, the reflectance was measured with a microspectrophotometer, which has an objective with a limited aperture. Incident light is reflected by the spherical facet lenses into a larger solid angle than is captured by the objective, the effect of which is even much worse for the reference, a diffuser. Another uncertainty is the variation in anatomical properties, as indicated by the slightly varying density profiles (Fig. 2c, d), meaning that the refractive index profiles vary across the facet lenses. Also the applied conversion of electron density into refractive index is a heuristic method. This data nevertheless formed a valuable basis for the optical modeling.

We estimated that the reflectance of the facet lenses at the peak wavelength is ~0.2, or, that the transmittance at that wavelength is ~0.8. This corresponds well with transmittance measurements performed on the green and red reflecting facets of the dolichopodid *Poecilobothrus nobilitatus* in isolated corneas by Knüttel and Lunau (1997). Larger reflectance peaks, and accordingly stronger drops in transmittance, were measured in the green facet lenses of the deerfly *Chrysops relictus* (Lunau and Knüttel 1995; Stavenga 2002).

### Spectral filtering to improve color vision?

The spectrally reduced transmittance causes the facet lenses to act as spectral filters. Trujillo-Cenóz and Bernard (1972) hypothesized that the spectral shifts induced by the corneal filters contribute to improving color contrast vision. Indeed, the modeling of Fig. 4 shows that the corneal filters can narrow the photoreceptor sensitivity spectra, depending on the relative position of the rhodopsin and transmittance spectra. Although the spectral changes are not large, they are possibly sufficient to have a beneficial effect on spectral discrimination.

To quantitatively predict the effect of the reduced transmittance of the two types of facet lenses on the underlying photoreceptors it is essential to know at least the visual and possibly other light-absorbing pigments of the photoreceptors. Unfortunately, very little is known of the spectral properties of the dolichopodid retina. We, therefore, have to rely on anatomical and physiological studies that established that the retinae of dolichopodids and higher brachycerans are similarly organized (Trujillo-Cenóz and Bernard 1972; Wunderer and Smola 1982; Hardie 1985). In the eyes of the fruitfly *Drosophila*, the R1–6 photoreceptors of all ommatidia express the rhodopsin Rh1, which absorbs maximally at 480 nm, and thus is also called R480, but the ommatidia differ concerning the rhodopsins expressed by the R7 and R8 photoreceptors (Salcedo et al. 1999). In the pale (p) type, the R7 and R8 photoreceptors express Rh3 (R331, absorbing in the UV) and Rh5 (R442, absorbing in the blue), respectively, and in the yellow (y) type the R7 and R8 express Rh4 (R355, absorbing in the UV) and Rh5 (R515, absorbing in the green).

In other higher brachycerans, the expression patterns of the photoreceptors are similar, but the visual pigment absorption spectra, and hence the photoreceptor spectral sensitivities, vary (Hardie 1985; Schmitt et al. 2005). In the housefly *Musca*, the rhodopsins in R7p, R7y, R8p and R8y are R335, R430, R460 and R520, respectively. The R7y rhabdomeres contain additionally to the visual pigment a blue-absorbing carotenoid pigment, which strongly modifies the spectral sensitivity of both the R7y and R8y photoreceptors. Notably, the R8y spectral sensitivity band is narrowed and shifted so that the peak wavelength becomes 540 nm (Hardie 1985). The R7y rhabdomeres of *Drosophila* contain also carotenoid pigment (Feiler et al. 1992), but whether this is also the case in dolichopodids remains unknown. Recordings from green sensitive R8 receptors in the tsetse fly *Glossina morsitans* yielded sensitivity spectra suggesting that at least there a carotenoid filter pigment is absent; the R1–6 photoreceptors had a peak sensitivity at ~500 nm (Hardie et al. 1989). The main (R1–6) photoreceptors of the drone fly *Eristalis tenax* were identified as distinctly blue-sensitive photoreceptors as the peak sensitivity is at 450–460 nm; additional UV and green receptors were also encountered (Horridge et al. 1975; Stavenga 1976; Tsukahara et al. 1977; Bernard and Stavenga 1979). The R1–6 receptors of other hover flies (*Syrphus* and *Allograpta*) have also blue-peaking sensitivity spectra. This holds for both the ventral and dorsal eye area of females, but in males the R1-6 of only the main ventral eye area are blue receptors, while in the dorsal area spectra with longer peak wavelength, like those in *Calliphora*, were found (Bernard and Stavenga 1979; Stavenga 1979). The R1-6 of the related soldier flies have peak sensitivities in the blue or green (Oonincx et al. 2016), but the R1-6 of other lower brachycerans, as for instance the horsefly *Tabanus nigrovittatus* have a clear green-peaking spectral sensitivity (Allan et al. 1991).

**Fig. 5:**
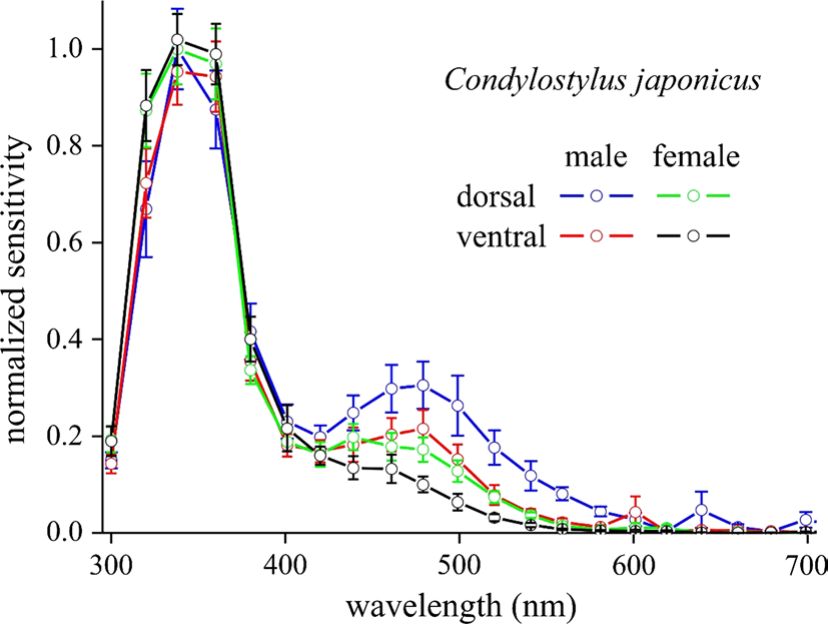
Normalized sensitivity spectra recorded by electroretinography from dorsal and ventral eye areas of both male (*n* = 6) and female (*n* = 6) long-legged flies *Condylostylus japonicus* (*error bars* indicate standard deviations)

Electroretinograms of male and female *Condylostylus*, measured from the dorsal and ventral eye areas, revealed a main ultraviolet component with a clear sideband in the blue-green and low responsivity in the green wavelength range (Fig. 5). The prominent UV band is presumably caused by the cumulative effect of the UV-sensitive R7 receptors and the UV sensitivity of the blue-green sensitive R1–6 receptors, enhanced by sensitizing pigment (Kirschfeld et al. 1983). The spectral sensitivities differ between the two sexes as well as the eye region, which is probably related to the regionalization of the facet colors (Bernard 1971). If we, nevertheless, may assume that the dolichopodids have R8 with similar spectral properties as those of the well-studied higher brachycerans it is a likely speculation that the facet lenses act as spectral filters for blue (R1–6, R8p) and green (R8y) photoreceptors.

### Relationship with polarization vision

Dolichopodid flies live among the leaves, which represent a very complex visual environment with a large diversity of polarized specular reflections from the foliage (Shashar et al. 1998). Polarization vision in flies is prominent in the dorsal rim, where pure UV receptors are specialized for detecting polarized skylight patterns. The ventral eye regions also mediate polarization vision, as was demonstrated for *Drosophila*, in which the central photoreceptors R7 and R8 as well as the peripheral receptors R1-6 are involved (Hardie 2012; Wernet et al. 2012). The R7 and R8 rhabdomeres of the two ommatidial classes in dipteran eyes have remarkable differences concerning the spatial orientation of the microvilli (Wunderer and Smola 1982). In the dolichopodid *Sympycnus lineatus*, the microvilli of the two R7 types are orthogonally arranged. All R8 photoreceptors and one class of R7 have vertical microvilli that are potentially blind to the specular reflections. The only type of R7 cells with horizontal microvilli resides in the green/yellow-faceted ommatidia. Possibly, polarization vision, based on the comparison of signals from R7 and R8, is enhanced by the facets’ spectral filters. Evidently, detailed electrophysiological experiments will be necessary to further clarify the filter actions of the dolichopodid facet lenses.

## Electronic supplementary material

Below is the link to the electronic supplementary material.

Supplementary material 1 (DOCX 559 kb)
