# Supplementary material for: Photoreceptor spectral tuning by colorful, multilayered facet lenses in long-legged fly eyes (Dolichopodidae)
Source: J Comp Physiol A Neuroethol Sens Neural Behav Physiol. 2016 Nov 21;203(1):23–33. doi: 10.1007/s00359-016-1131-y (PMC5263225; doi:10.1007/s00359-016-1131-y)
Supplement: Supplementary file 1 — Supplementary material 1 (DOCX 559 kb) [file 359_2016_1131_MOESM1_ESM.docx]

**Supplementary figures**

**
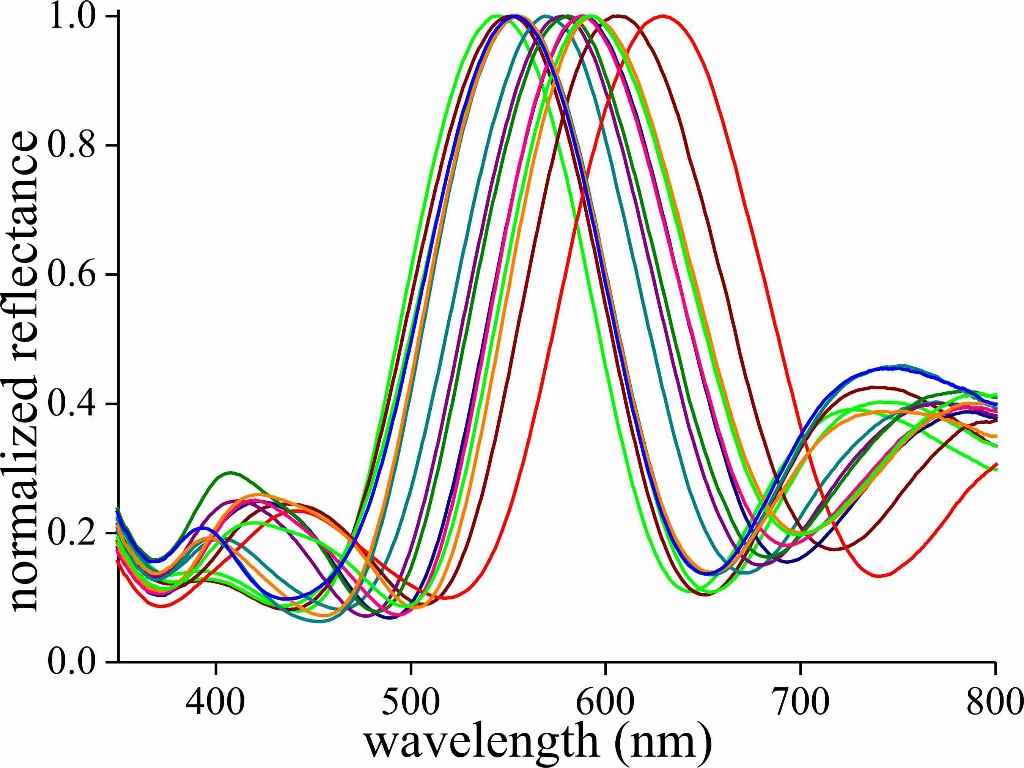
**

Fig. S1. Normalized reflectance spectra of several adjacent facet lenses of *Dolichopus nitidus*, measured with a microspectrophotometer, showing a virtually identical spectral shape.


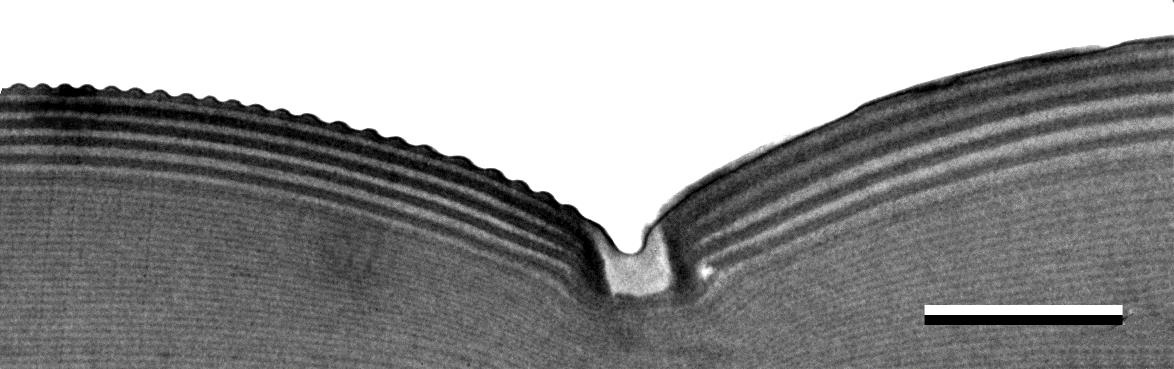


Fig. S2. Transmission electron micrograph of two adjacent facet lenses with layer period of 176 nm (left) and 224 nm (right); scale bar: 2 µm.

**
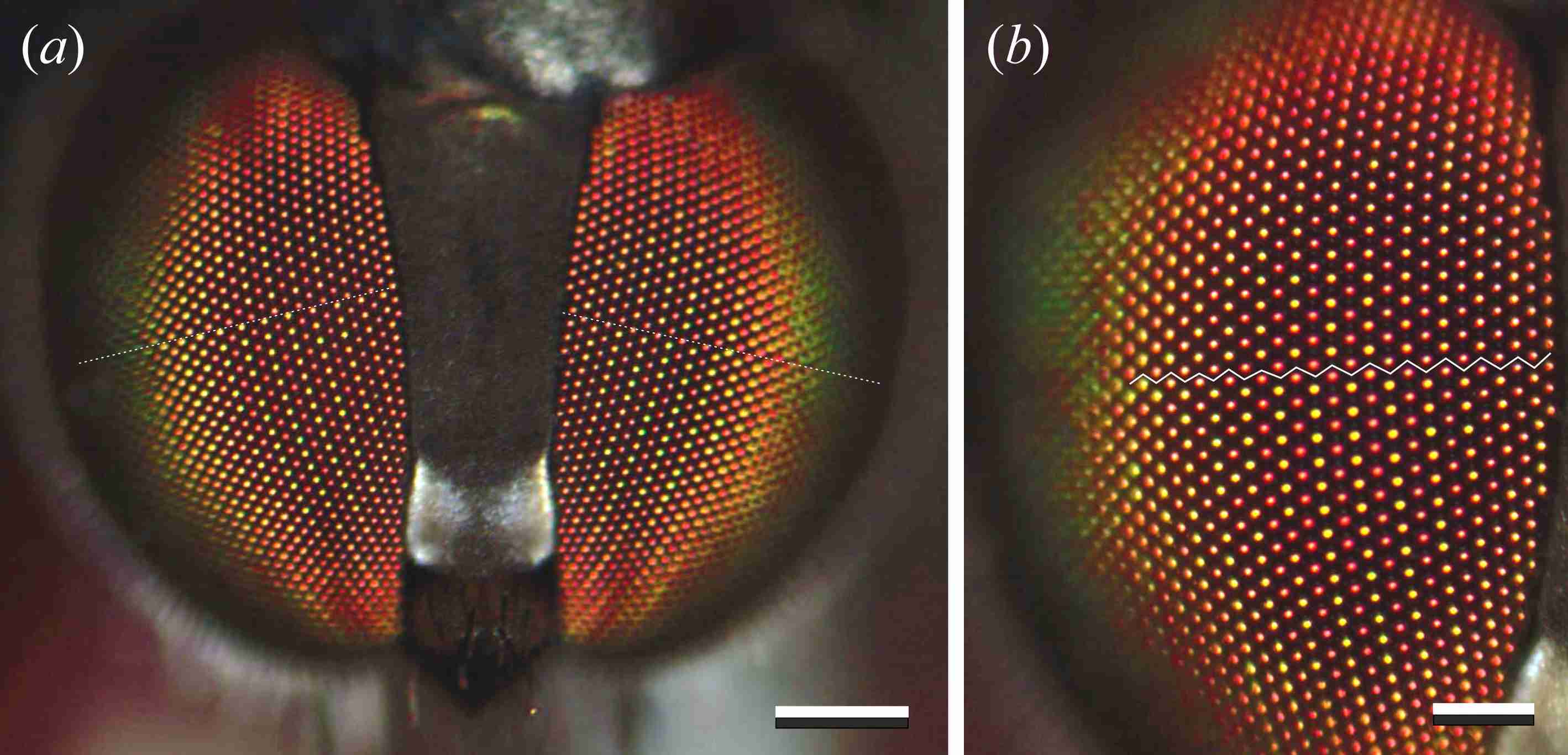
**

Fig. S3. An unidentified long-legged fly with rows of green-yellow and orange-red facets (captured 1 August 2016 in Haren, Groningen, Netherlands). The pattern of colored rows shows an abrupt shift at the indicated lines. Bars: (*a*) 200 µm, (*b*) 100 µm. Note that the cuticle has no metallic reflection.
